# Generation of induced cardiac progenitor cells *via* somatic reprogramming

**DOI:** 10.18632/oncotarget.15272

**Published:** 2017-02-10

**Authors:** Jianyong Xu, Wei Lian, Lingyun Li, Zhong Huang

**Affiliations:** ^1^ Institute of Biological Therapy, Shenzhen University, Shenzhen, China; ^2^ Department of Pathogen Biology and Immunology, Shenzhen University School of Medicine, Shenzhen, China; ^3^ Shenzhen City Shenzhen University Immunodiagnostic Technology Platform, Shenzhen, China

**Keywords:** cardiac progenitor cell, CPC, somatic reprogramming, induced CPC

## Abstract

It has been demonstrated that cardiac progenitor cells (CPCs) represent a more effective cell-based therapy for treatment of myocardial infarction. Unfortunately, their therapeutic application is limited by low yield of cell harvesting, declining quality and quantity during the ageing process, and the need for highly invasive heart biopsy. Therefore, there is an emerging interest in generating CPC-like stem cells from somatic cells *via* somatic reprogramming. This novel approach would provide an unlimited source of stem cells with cardiac differentiation potential. Here we would firstly discuss the different types of CPC and their importance in stem cell therapy for treatment of myocardial infarction; secondly, the necessity of generating induced CPC from somatic cells *via* somatic reprogramming; and finally the current progress of somatic reprogramming in cardiac cells, especially induced CPC generation.

Cardiovascular diseases (CVD) are the most prevalent diseases in the world and are associated with significant morbidity and mortality [1]. Myocardial infarction (MI) due to blockade of coronary arteries causing myocardial injuries is the most common cause of CVD. After MI, there is progressive cardiac remodelling, which can lead to left ventricular dilatation and heart failure [2].

Cell-based therapy has been proposed as a promising strategy for treating MI and adverse heart remodelling. Transplantation of healthy and functional cells would replenish the damaged cells and repair the injured heart [3]. Different types of cells, including skeletal myoblasts, bone marrow stem cells (BMSCs), mesenchymal stem cells (MSCs), endothelial progenitor cells (EPCs), cardiomyocytes, and cardiac progenitor cells (CPCs), have been studied for treating MI [4–8]. Because of the potential arrhythmia risk of skeletal myoblasts and cardiomyocytes for treating MI, they are not discussed here [9, 10]. BMSCs, MSCs and EPCs have been demonstrated effective for treating MI. However, their direct involvement in cardiac regeneration with cardiomyocyte differentiation is controversial [6]. On the other hand, CPCs are safer and more effective compared to BMSCs, MSCs and EPCs for treating MI, with evidences of direct cardiac differentiation [6].

## CARDIAC PROGENITOR CELLS

**Figure 1:**
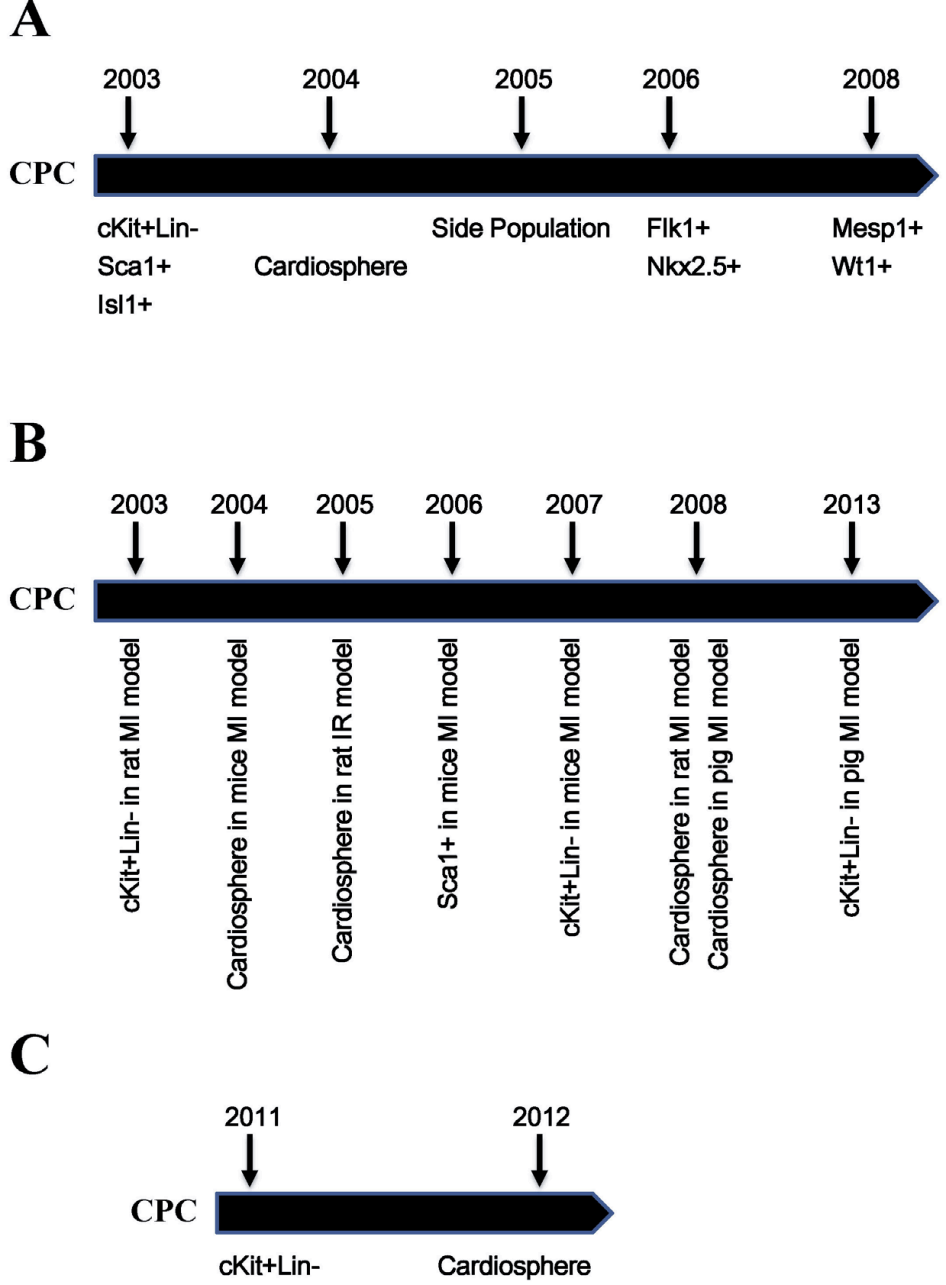
Timeline of the discovery of CPCs and their applications **A**. Timeline of the discovery of different populations of CPCs. **B**. Timeline of the pre-clinical studies of CPCs. **C**. Timeline of the clinical studies of CPCs. CPC: cardiac progenitor cell; MI: myocardial infarction; IR: ischemia reperfusion.

CPCs (Cardiac Progenitor Cells) are localised in the heart. They have the abilities of self-renewing and differentiating into cardiomyocytes, endothelial cells and smooth muscle cells (the three major cell types of the heart) [11, 12]. CPCs have become an important player in cardiac homeostasis under both physiological (continual cellular turnover) and pathological (proliferative activity and regenerative potential) conditions. Since the first demonstration of CPCs as the c-Kit^+^Lin^−^ population [11], different kinds of CPCs have been identified (Figure 1, Table 1), including Flk1^+^ [13, 14], Sca1^+^ [15], side population [16, 17], Mesp1^+^ [18, 19], Isl1^+^ [20–22], Nkx2.5^+^ [23], Wt1^+^ [24, 25] and cardiospheres [26].

**Table 1:** Comparison of resident cardiac progenitor cells

Cell Type	Markers	Developmental Origin	Self Renewal	Clonality	Differentiation Potential	Functional Characterization *in vitro*	Function *in vivo*	Reference
cKit+Lin-	cKit+; Nkx2.5low; Gata4low; Mef2clow; CMC marker low; CD34-; CD45-; CD20-; CD8-; EC Marker-; SMC Marker-; Lin-	ND	Yes	Yes	CMC; SMC; EC	CMC markers; sarcomere markers; action potential	Function improvement of the infarcted heart with regeneration	[11]
Flk1+	Flk1+; Mesp1+; Isl1+; Nkx2.5+	ND	Yes	Yes	CMC; SMC; EC	CMC markers; sarcomere markers; action potential; spontaneous contraction	ND	[13; 14]
Sca1+	Sca1+; Gata4+; CD38+; CD31+; Mef2c+; CD34-; Nkx2.5-; cKit-; Flk1/Flt1-; CD45-; vWF-; Lin-; CMC Marker-	ND	ND	ND	CMC	CMC markers; sarcomere markers	Engrafted into the infarcted myocardium	[15]
Side Population	Abcg2+; Sca1+; CD31+; Mef2c+; cKit-; CD45-; CD34-; Nkx2.5-; Gata4-; CMC Marker-	ND	Yes	Yes	CMC	CMC markers; sarcomere markers; calcium transient; spontaneous contraction	Engrafted into the infarcted myocardium	[16, 17]
Mesp1+	Mesp1+; Nkx2.5+; Hand2+; Gata4+; CXCR4+; Flk1+; PDGFRa+	Mesp1+ cells contribute to the whole heart development by lineage tracing analysis	Yes	Yes	CMC; SMC; EC	CMC markers; sarcomere markers;	Engrafted into the infarcted myocardium	[18, 19]
Isl1+	Isl1+; Nkx2.5+;Gata4+; Sca1-; cKit-; CD31-; CMC Markers-; SMC markers-	Isl1+ cells contribute to the second heart field development by lineage tracing analysis	Yes	Yes	CMC; SMC; EC	CMC markers; sarcomere markers; action potential; calcium transient; spontaneous contraction	ND	[20, 21, 22]
Nkx2.5+	Nkx2.5+; Isl1+; cKit+; Sca1+; Lin-; EC Marker-	Nkx2.5+ cells contribute to the whole heart development by lineage tracing analysis	Yes	Yes	CMC; SMC	CMC markers; sarcomere markers; action potential; spontaneous contraction	Engrafted into the infarcted myocardium	[23]
Wt1+	Wt1+; Isl1+; Nkx2.5+	Wt1+ cells contribute to the whole heart development by lineage tracing analysis	ND	ND	CMC; SMC; EC	CMC markers; sarcomere markers; action potential; calcium transient; spontaneous contraction	Engrafted into the infarcted myocardium	[24, 25]
Cardiosphere	Flk1+; cKit+; CD34+; Sca1+; vWF+; CD31low; cTnT+; MHC+; CD105+	ND	Yes	Yes	CMC; SMC; EC	CMC markers; sarcomere markers; action potential; calcium transient; spontaneous contraction	Function improvement of the infarcted heart with regeneration	[26]

CMC: Cardiomyocyte; SMC: Smooth Muscle Cell; EC: Endothelial Cell; ND: Not Determined

### c-Kit^+^Lin- population

The first report of purification and characterisation of CPCs was published in 2003 and defined as c-Kit^+^Lin^−^ cells [11]. To prove the existence of adult CPCs in the rat heart, Antonio et al. analysed three stem cell surface markers, including c-Kit, Sca1, and Flk1, which are commonly expressed by other adult stem cells. Immunohistological analysis showed that some c-Kit^+^Lin^−^, Sca1^+^Lin^−^, and Flk1^+^Lin^−^ cells localised in the heart with a high nucleus/cytoplasm ratio. Finally, they focused on the c-Kit^+^Lin^−^ population for further investigation because of the therapeutic effect of the c-Kit^+^Lin^−^ cell population in treating MI [27]. The purified c-Kit^+^Lin^−^ cells were positive for cardiac markers Nkx2.5 and MEF2C, but negative for leukocyte marker CD45 and the hematopoietic progenitor marker CD34. They were self-renewing, clonogenic, and multi-potent, with the ability to differentiate into cardiomyocytes, endothelial cells, and smooth muscle cells both *in vitro* and *in vivo*. The cardiac regeneration role of c-kit^+^Lin^−^ cells in the animal model was proven to not result from cell fusion [11].

### Flk1^+^ population

By using the GFP (Green Fluorescent Protein)-Bry mouse embryonic stem cell (mESC) line, in which GFP is expressed under the brachyury (Bry) promoter, it has been demonstrated that a GFP^+^/Flk1^+^ cell population during mESC differentiation has the ability to differentiate into beating cardiomyocytes *in vitro*. GFP^+^/Flk1^+^ cells express cardiac progenitor markers (Mesp1, Isl1, and Nkx2.5) with clonogenic capability and can also be differentiated into endothelial cells and smooth muscle cells. To determine whether this cell population exist *in vivo*, different regions and different stages of mouse embryos were cultured *in vitro*, and it was found that the colonies generated from anterior neural plate embryos and head-fold-stage embryos could become beating colonies. Of these, the head-fold-stage embryos produced more beating colonies. Then, the Flk1^+^ cells were purified from the head-fold-stage embryos and they had the ability to differentiate into cardiomyocytes, endothelial cells, and smooth muscle cells [13, 14]. This strategy was also applied to human ESC and a similar Flk1^+^ cell population with cardiac differentiation abilities (cardiomyocytes, endothelial cells, and smooth muscle cells) was discovered [13, 14].

### Sca1^+^ population

Oh et al. analysed the stem cell markers Sca1 and c-Kit in adult mouse cardiac cells, in which the cardiomyocytes were depleted *via* enzyme digestion. It was found that 14-17% of them were Sca1^+^. The Sca1^+^ cells did not express blood cell lineage markers, hematopoietic stem cell markers, or endothelial cell markers. They expressed cardiogenic genes, but not mature cardiac structural genes. After cardiomyocyte differentiation, they started to express mature cardiac structural genes. By using a lineage tracing system, the transplanted Sca1^+^ cells were recruited into the infarct region in a mouse MI model and expressed cardiomyocytes markers [15].

### Side population

Side population cells are defined by their capacity to efflux Hoechst dye through an ATP (Adenosine Triphosphate)-binding cassette transporter. After depleting the cardiomyocytes, there was a population of Hoechst-low cells existing in the mouse heart-derived cells. The cardiac side population cells are capable of self-renewal and differentiating functional cardiomyocytes with spontaneous contracting [16, 17]. And the Hoechst efflux ability of cardiac side population cells was completely inhibited by the ATP-binding cassette transporter inhibitor. They were negative for CD45, CD34, CD44, and c-Kit, but positive for CD31 and Sca1. The cardiac side population cells formed colonies, indicating their multi-potency characteristics. And their cardiomyocytes derivatives coupled with adult cardiomyocytes *in vitro via* the co-culture system without cell fusion events [16, 17]. Under physiologic conditions, the cardiac side population cells maintained their cell pool through cell proliferation without recruiting extra-cardiac stem cells. After MI, the cardiac side population cells were depleted quickly, and then the cell pool was reconstituted later through cell proliferation and recruiting stem cells from bone marrow [16, 17].

### Mesp1^+^ population

Mesp1 is the earliest marker in heart development, and almost all of the heart and related vessels are developed from the Mesp1^+^ cells through lineage tracing studies [18, 19]. Transient expression of Mesp1 dramatically enhanced CPC generation and also cardiomyocyte differentiation in mouse ESC. Through whole-genome expression profiling and chromatin immunoprecipitation (ChIP) analysis, it has been shown that Mesp1 could directly upregulate cardiac transcription factors, such as Hand2 and Nkx2.5, and the Wnt pathway. In addition, Mesp1 suppressed the expression of genes related to pluripotent, endoderm, and early mesoderm [18, 19]. Then, the ESC cell line with GFP expression driven by the Mesp1 promoter was established to purify the Mesp1^+^ cells. The purified Mesp1^+^ cells enriched CPCs with abilities to differentiate into cardiomyocytes, endothelial cells, and smooth muscle cells. Transplanting these Mesp1^+^ cells into the kidney capsule of immunodeficient mice showed that they mainly differentiated into cardiomyocytes *in vivo* and, to a lesser extent, endothelial cells and smooth muscle cells [18, 19].

### Isl1^+^ population

Isl1 is a transcription factor modulating heart development; lack of Isl1 results in heart abnormalities [20–22]. Using a lineage tracing strategy, the Isl1^+^ cells represent a new population of CPCs involved in heart development. Approximately 30-40% cardiomyocytes originated from Isl1^+^ cells during heart development. Purified Isl1^+^ cells showed functional ability of cardiomyocyte differentiation [20–22]. Using the mouse ESC cell line, Isl1^+^ cells were further proven as a CPC population with the ability to differentiate in cardiomyocytes, endothelial cells, and smooth muscle cells [20–22].

### Nkx2.5^+^ population

By using transgenic mice with GFP expression driven by the cardiac-specific Nkx2.5 enhancer, it was demonstrated that Nkx2.5 expression overlapped partially with Isl1 and completely overlapped with the sarcomeric myosin heavy chain [23]. Isolated Nkx2.5^+^ cells from embryos showed cardiomyocyte, conduction system cell, and smooth muscle cell differentiation ability. Purified Nkx2.5^+^ cell during mouse ESC differentiation also showed cardiomyocyte and smooth muscle differentiation ability *in vitro* and *in vivo* [23]. These cells were positive for c-Kit and Sca1, but negative for hematopoietic and endothelial markers [23]. Later study also showed that NKX2.5 positive CPCs could be generated from human ESC [28].

### Wt1^+^ population

By knocking-in GFP after the gene Wt1 (Wilms tumour 1), it was demonstrated that one population of CPCs located within the epicardium expressed the transcription factor Wt1. The data showed that some of the Wt1^+^ cells migrated and differentiated into functional cardiomyocytes during heart development. The cardiomyocytes originated from Wt1^+^ progenitor cells were located in all four chambers of the heart. Furthermore, these progenitor cells originated from early CPCs that expressed Nkx2.5 and Isl1. The purified Wt1^+^ cells also had the capability of differentiating into beating cardiomyocytes, endothelial cells, and smooth muscle cells [24, 25]. The Wt1^+^ CPCs were activated after MI or thymosin beta4. Transplanting these Wt1^+^ cells into the heart after MI showed functional cardiomyocyte differentiation and integration into the resident myocardium [24, 25].

### Cardiosphere

Cardiospheres are composed of sphere-forming cells isolated from human heart biopsy (atrial and ventricular) and mouse heart (embryo, fetal, and postnatal). These sphere-forming cells originate from small, round, and phase-bright cells that migrated from the heart explants [26]. Cardiospheres generated from mouse heart could beat spontaneously after sphere formation. However, the human cardiospheres did not have this capability unless they were co-cultured with rat neonatal cardiomyocytes. Cardiospheres could attach onto fibronectin-coated plates. They formed spheres when growing on poly-D-lysine-coated plates. They contained Flk1, CD31, CD34, c-Kit and Sca1 positive cells. When transplanting these cardiospheres into the dorsal subcutaneous region or the heart after MI, they had the capability to differentiate into cardiomyocytes, endothelial cells, and smooth muscle cells *in vivo* [26]. Although the cardiac stem cell property of cardiospheres has been questioned [29], the cardiosphere concept has been widely accepted.

## PRE-CLINICAL AND CLINICAL STUDIES OF CARDIAC PROGENITOR CELLS

The therapeutic potential of CPCs has been intensively studied in animal models [30]. And the safety and practicability are further demonstrated by clinical studies. Among all types of CPCs, only two of them are widely studied for their therapeutic application in treating MI, c-Kit^+^ CPCs and cardiosphere-derived cells [31] (Figure 1, Table 2, Table 3 and Supplementary Table 1).

**Table 2:** Pre-clinical studies of CPC based cell therapy in heart diseases

Cell source	Animal model/Species	Cell number	Route of delivery	Outcomes	Mechanism	Reference
Human cKit+ CPC	MI/mice and rat	4×10^4^	Intramyocardial	Attenuation of chamber dilation, improvement of ventricular function	CMC, EC, SMC differentiation	[31]
Autologous cKit+ CPC	IR/pig	5×10^5^	Intracoronary	Improved LVEF, reduced LV end-diastolic pressure	CMC, EC, SMC differentiation	[32]
Human Cardiosphere	MI/mice	1×10^5^	Intramyocardial	Improved LVEF	CMC, EC, SMC differentiation	[43]
Autologous Cardiosphere	MI/pig	1×10^7^	Intracoronary	Decreased infarct size, improved dP/dt	CMC differentiation	[37]
cKit+ CPC overexpressing Pim-1	MI/mice	1×10^5^	Intramyocardial	Reduced infarct size, increased vasculature, improved LVEF	Recruitment of endogenousstem cells; increased CMC proliferation; decreased apoptotic cell death; CMC, EC, SMC differentiation	[33]
Human cardiosphere	MI/mice	10 spheres/animal	Intramyocardial	Preserved wall thickness, improved fractional shortening	CMC, EC, SMC differentiation	[26]
Rat cardiosphere	IR/rat	1×10^6^	Intracoronary	Improved left ventricular function, reduced fibrosis	Proliferation of endogenous CPCs; CMC, EC, SMC differentiation	[45]
Mouse cardiosphere	MI/mice	2×10^5^	Intramyocardial	Decreased scar size, increased viable myocardium, improved cardiac function	Stimulated resident cardiomyocyte cycling; recruitment of endogenous CPC; CMC, EC, SMC differentiation	[40]
Rat cardiosphere	MI/rat	2×10^6^	Intramyocardial	Reduced scar size and collagen content, increased in viable mass, improved left ventricular function	Increased expression of the regenerative growth factors: VEGF, HGF and insulin-like growth factor-1; stimulated angiogenesis; attenuated inflammatory response; reduced proinflammatory cytokines	[46]
Human cardiosphere	MI/mice	1×10^5^	Intramyocardial	Improved LVEF	Secreted VEGF, HGF and insulin-like growth factor 1; increased the expression of Akt; decreased apoptotic rate and caspase 3 level; increased capillary density; CMC, EC, SMC differentiation; recruiting endogenous CPC and improving tissue resistance to ischemic stress	[35]
cKit+ CPC	MI/rat	1×10^5^	Intramyocardial	Reduced Infarct size, increased capillaries density, improved LVEF, reduced cavitary dilation, increased wall thickness, improved LV ejection fraction and dP/dt	CMC, EC, SMC differentiation	[11]
Rat Cardiosphere	IR/rat	1×10^6^	Intracoronary	Attenuated LV dilation, increased wall thickness, decreased infarct size	SMC EC differentiation	[36]
Rat Cardiosphere	MI/rat	4×10^4^	Intramyocardial	Attenuated ventricular dilation, prevented the chronic decline in function, improved LVEF	CMC differentiation, synthesize matrix metalloproteinase, cytokines secretion	[79]
Autologous Cardiosphere	MI/pig	1×10^7^	Intramyocardial	Improved ejection fraction, attenuated adverse remodeling	Not addressed	[38]
Human Cardiosphere	MI/mice	1×10^5^	Intramyocardial	Improved LVEF and wall thickness, reduced infarct size	CMC, EC, SMC differentiation; reduced apoptotic cells; elevated cytokines secretion	[39]
Human Cardiosphere	MI/pig	2×10^7^	Intramyocardial	Improved LVEF, reduced infarct size	heart regeneration, CMC differentiation	[44]
Mouse cKit+ CPC	Acute heart failure/mice	5×10^5^	Tail-vein	Improved LV fraction shortening	cardiac regeneration, CMC differentiation	[34]
Mouse Sca1+/CD31- CPC	MI / mice	1×10^6^	Intramyocardial	Attenuated adverse structural remodeling, increased LVEF, increased neovascularization	CMC, EC differentiation	[80]
Human cardiosphere	MI / mice	1×10^7^	Intramyocardial	Preserved myocardial function, prevented adverse remodeling, and enhanced blood vessel preservation	CMC, EC differentiation	[42]

CPC: cardiac progenitor cell; MI: myocardial infarction; IR: ischemia re-perfusion; LV: left ventricular; LVEF: left ventricular ejection fraction; CMC: cardiomyocyte; EC: endothelial cell; SMC: smooth muscle cell

**Table 3:** Clinical studies of CPC-based cell therapy in heart diseases

Cell source	Cell number	Disease	Route of delivery	Outcomes	Mechanism	Reference
Autologous cKit+ CPC	5-10×105	MI (*n* = 20)	Intracoronary	Improved LVEF, decreased infarct size at 4 months and 1 year	Not addressed	[51]
Autologous cKit+ CPC	1×106	MI (*n* = 16)	Intracoronary	Improved LVEF, decreased infarct size at 1 year	Not addressed	[52]
Autologous cardiospheres	2-3×106	Hypoplastic left heart syndrome (*n* = 7)	Intracoronary	Improved LVEF, reduced heart failure status and increased viable tissue at 18 and 36 months	Not addressed	[53, 56]
Autologous cardiospheres	12.5-25×106	MI (*n* = 17)	Intracoronary	Improved LVEF, decreased scar mass and increased viable tissue at 1 year	Not addressed	[54, 55]

CPC: cardiac progenitor cell; MI: myocardial infarction; LVEF: left ventricular ejection fraction.

Transplanting the c-Kit^+^ CPCs into animal models with injured hearts promoted myocardial regeneration, heart function improvement, adverse heart remodelling attenuation, and cell death reduction with cardiomyocytes differentiation (Table 2) [11, 32–35]. In addition to the role of improving heart function and cardiac cell differentiation, transplanting cardiospheres or cardiosphere-derived cells also showed the effects of anti-apoptotic, anti-fibrotic, activating endogenous CPCs, cytokine secretion, and inflammation modulation (Table 2) [26, 36–47]. Furthermore, transplanting the cardiospheres in the form of the sphere was more effective than the cardiosphere-derived cells, indicating that the three-dimensional structure maintained the niche for stem cells [48]. Combing the MSCs with cardiosphere-derived cells also showed increased efficacy in improving heart function and cardiac regeneration [49–51].

Because of the promising results in pre-clinical animal models, clinical trials were conducted to assess the safety, efficacy, and feasibility of CPCs in treating patients (Table 3 and Supplementary Table 1) [52–57]. The first phase 1 clinical trial using CPCs was conducted to investigate their safety and feasibility. Autologous c-Kit^+^/Lin^−^ CPCs were harvested during coronary artery bypass graft surgery and then used to treat heart failure patients with intracoronary infusion. Heart function was improved as early as 4 months after cell transplantation, and this effect continued up to 1 year later. During this period, the control group did not show any evidence of functional improvement. Furthermore, after CPCs transplantation, infarct size decreased, ventricular mass increased, and no adverse effects were observed (Table 3) [52, 53].

Transplanting autologous cardiospheres also showed cardiac function improvement, scar size reduction without any adverse events and tumour formation, indicating their safety and feasibility (Table 3) [54–56]. The ongoing clinical trials are designed to further address the effectiveness, cell type, cell number, delivery strategy, time window, and other factors (Table 4).

**Table 4:** Generation of induced CPC *via* somatic reprogramming

Purification Approach	Starting Cell Type	Transcription Factors	Growth Factors or Chemicals	CPC Marker Expression	Whole Genome Gene Expression Profile	Cardiac Differentiation *In Vitro*	Differentiated Cardiomyocytes Characterization	Cardiac Differentiation *In Vivo*	Therapeutic Application in MI model	Tumor Formation	References
Cardiosphere	MEF, AEF	Sox2, Klf4, Oct4	BIO, OSM	Mesp1, Isl1, Nkx2.5	Clustered with endogenous cardiosphere	CMC, EC, SMC	Action potential, Calcium transient, Contractility	CMC, EC	Improved heart function, reduced infract size, increased capillary density	Not detected in 12 weeks	[129]
Flk1^+^PdgfR ⍺*	MEF, TTF	Sox2, Klf4, Oct4, c-Myc	BMP4, Activin A, CHIR99021, SU5402	Flk1^+^PdgfR ⍺ ^+^	Clustered with ESC derived CPC	CMC, EC, SMC	Action potential, Calcium transient, Contractility	CMC, EC, SMC	Improved heart function,	Not analyzed	[128]
Nkx2.5^+^	CF, LF, TTF	Mesp1, Tbx5, Gata4, Nkx2.5, Baf60c	LIF, BIO	Nkx2.5, Irx4	Clustered with ESC derived CPC	CMC, EC, SMC	CMC marker expression without beating	CMC, EC, SMC	Improved survival	Not detected in 4 weeks	[130]

MEF: mouse embryonic fibroblast; AEF: adult mouse ear fibroblast; TTF: mouse tail-tip fibroblast; CF: mouse cardiac fibroblast; LF: mouse lung fibroblast; OSM: oncostatin M; ESC: mouse embryonic stem cell; CPC: cardiac progenitor cell; CMC: cardiomyocyte; EC: endothelial cell; SMC: smooth muscle cell.

## MECHANISMS OF CELL THERAPY AND THE KEY ROLE OF CARDIAC PROGENITOR CELLS

**Figure 2:**
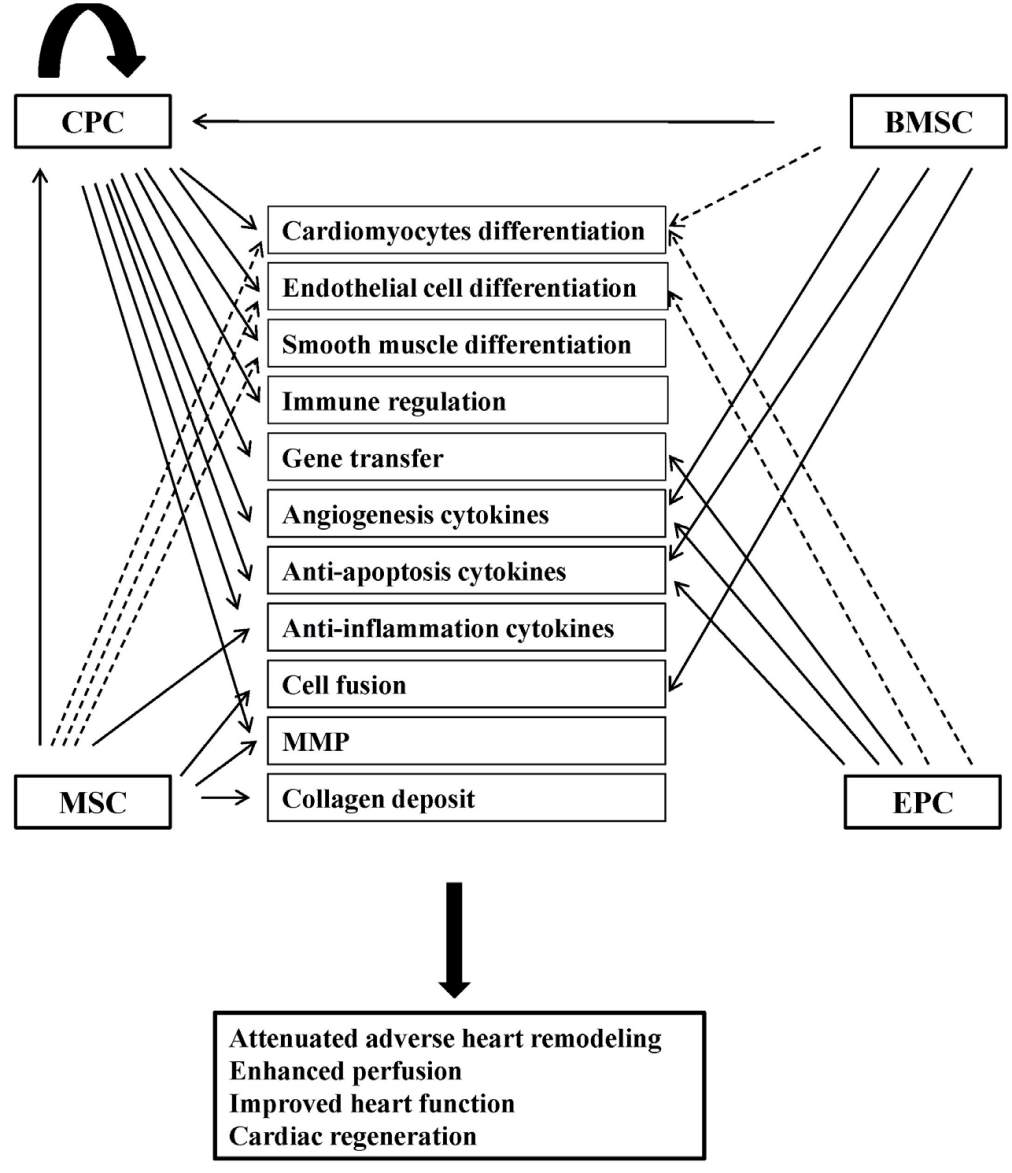
Potential mechanisms of stem cell therapy for myocardial infarction The potential mechanisms have been proposed as direct cardiac differentiation (cardiomyocytes, endothelial cells and smooth muscle cells), paracrine effects (immune regulation, gene transfer, angiogenesis cytokines, anti-apoptosis cytokines, anti-inflammation cytokines, MMP, collagen deposit), and cell fusion. CPC: cardiac progenitor cell; BMSC: bone marrow stem cell; MSC: mesenchymal stem cell; EPC: endothelial progenitor cell; MMP: matrix metalloproteinase. Dash lines indicate that the evidences are controversial.

Two goals should be achieved in MI treatment. The first is to prevent cardiomyocyte death and adverse heart remodelling. The second is to promote cardiac repair or regeneration and preserve and recover cardiac function. So far, the potential mechanisms of stem cell therapy for treating MI have been proposed as direct cardiomyocyte differentiation, blood vessel formation (endothelial and smooth muscle cell differentiation), cell fusion, and paracrine effects (endogenous CPC activation, neovascularisation, and apoptosis inhibition) (Figure 2) [7, 58].

The efficacy of BMSCs, MSCs, and EPCs in treating myocardial infarction is evident. However, the underlying mechanism is believed to be paracrine effects and cell fusion, but not direct cardiomyocyte differentiation [58–63]. The paracrine effects include promoting angiogenesis [64–66], preventing apoptosis [67, 68], suppressing inflammation [69–73], modulating extracellular matrix dynamics [74] and transferring genes into the local cardiomyocytes [75]. However, there are some other studies that have argued that direct cardiomyocyte differentiation also contributes to improvement in heart function [42, 76–79].

CPCs also have shown effectiveness in improving heart function, increasing neovascularisation, reducing infarct size, and attenuating adverse remodelling. Differing from BMSCs, MSCs and EPCs, for which the paracrine effect is the main mechanism of heart function improvement, it has shown strong evidences of direct cardiac differentiation (cardiomyocytes, endothelial cells, and smooth muscle cells) of CPCs [11, 26, 32–41, 43–47, 80, 81]. Furthermore, cardiosphere transplantation also shows paracrine effects (Table 2). For example, they secrete cytokines (such as vascular endothelial growth factor, hepatocyte growth factor and insulin-like growth factor), activate endogenous stem cells, decrease cell apoptosis, remodel extracellular matrix, and inhibit inflammation response [34, 36, 40, 41, 46, 47, 80].

In a rat MI model, the c-Kit^+^ CPCs migrated into the infarct region through collagen type I and type III bundles. Analysis of matrix metalloproteinases (MMP; responsible for degrading extracellular matrix) and its tissue inhibitors of MMP (TIMP) showed that CPCs transplantation strongly promoted the expression level of MMP2, MMP9, and MMP14, and inhibited TIMP4 expression. These data indicated that CPCs have invasive ability through modulating extracellular matrix [80]. Transplanted CPCs (c-Kit^+^ CPCs and cardiospheres) continued to proliferate *in vivo* and activated endogenous CPCs [36, 41, 46]. Overexpression of Pim1 in c-Kit^+^ CPC (component of AKT pathway that showed cardiac-protective activity) further enhanced the activity of CPCs [34]. Furthermore, miRNA could be transferred from CPC to cardiomyocytes [82–86].

MI triggers the resident cardiomyocytes to re-enter the cell cycle, up-regulating cell cycle-related genes. This effect was further amplified by cardiosphere transplantation [41]. Cardiosphere transplantation reduced pro-inflammatory cytokines (tumour necrosis factor-α, interferon-γ, interleukin-6, and interleukin-1β), which are normally activated by MI, and produced regenerative growth factors (VEGF, Vascular Endothelial Growth Factor; HGF, Hepatocyte Growth Factor; and insulin-like growth factor) [36, 47]. In a rat ischemia reperfusion (IR) model, cardiosphere transplantation was effective in reducing heart injury only when the transplantation was conducted in 20 minutes, but not after 2 days [87]. Cardiospheres could reduce the infarct size and prevent cardiomyocyte death through reducing pro-inflammatory factor secretion and the number of CD68^+^ macrophages. Cardiosphere cells activated the macrophage polarization, shifting them away from M1 macrophages, resulting in a reduction of the inflammation response. Cardiospheres that activated M2 macrophages polarization showed anti-oxidative and anti-apoptotic effects, and transplanting them into the rat IR model showed a reduction in heart injury [87]. More interestingly, the cardiospheres had been shown to be much more effective than c-Kit^+^ CPC for improving heart function because they secreted more cytokines [40].

CPCs in cell therapy for MI are important not only because they could differentiate into cardiac cells (cardiomyocytes, endothelial cells, and smooth muscle cells) and are involved in cardiac function preservation and recovery but also because they mediate the efficacy of other types of stem cells (BMSCs and MSCs, Figure 2). Therefore, CPCs are the most important player in heart function recovery after MI [35]. MSCs and BMSCs could stimulate endogenous CPC proliferation and differentiation, and the activated CPCs contribute to cardiac function improvement after MI [88, 89]. The cell mixture of MSCs and CPCs had a synergistic effect on treating MI [50]. And the cell hybrids through cell fusion of cardiac progenitors and MSCs showed better efficacy in myocardial repair. However, abolishing endogenous CPCs would eliminate the regeneration process [49].

Therefore, it is becoming much more obvious that the therapeutic effects of cell therapy for preventing adverse heart remodelling and promoting cardiac regeneration might work through cardiac progenitor activity. Accounting for cardiac differentiation abilities, the CPCs play a central role in cell therapy of MI (Figure 2).

## ENDOGENOUS CARDIAC PROGENITOR CELLS ARE NOT SUFFICIENT FOR MYOCARDIAL REGENERATION

The fact that most survivors of MI would eventually develop and die from congestive heart failure makes it clear that although there is some level of cardiomyocyte turnover in the adult heart, it is not sufficient to compensate for the cell loss [63]. In mice, cardiomyocyte regeneration occurs at a very low rate, and this is decreased during ageing and increased after injury [90]. Furthermore, both cell number and cell activity of cardiomyocytes and CPCs decline during ageing [91]. Cardiospheres derived from neonatal human atrium are more cardiomyogenic and more effective in the improvement of heart function in the mouse MI model [43].

Therefore, the body could not regenerate the heart by itself after the MI. After MI, the heart is in a high-inflammatory, fibrotic, low-nutrient, and hypoxia environment, which impairs the function and efficacy of cardiomyocytes, bone marrow stem cells, and local CPCs. Isolated CPCs from the heart could be used for transplantation after expansion *in vitro*. Considering that the procedures for cardiac cell harvesting are invasive and would cause more injury to patients with MI, the generation of induced CPCs *via* somatic reprogramming from somatic cells, which are more easily available, is necessary.

## CELL FATE CONVERSION VIA SOMATIC REPROGRAMMING IN CARDIAC CELLS

The embryo is developed in a temporal and spatial manner and is precisely controlled by cytokines and genes. Stem cells with differentiation abilities play a crucial role in the development process. Normally, the differentiation ability of stem cells would become more and more committed during development, and this process is difficult to reverse. Eventually, cells become terminally differentiated in different tissues with different characteristics and functions. This process is called cell fate determination. Long ago, people believed that once the cell fate is determined, it is difficult to change. However, this has been challenged with the development of somatic reprogramming technologies. Somatic reprogramming is used to convert the terminally differentiated somatic cells to a more progenitor cell state or even pluripotent stem cells state with more differentiation abilities [92–95]. After the breakthrough in somatic reprogramming with the development of induced pluripotent stem cell (iPSC) technology, the somatic reprogramming field has grown rapidly [96, 97]. iPSC are generated through overexpressing transcription factors (Sox2, Klf4, Oct4, and c-Myc), which are important for pluripotent stem cell maintenance, in terminal differentiated somatic cells, and this converts the somatic cells into pluripotent stem cells [98–101].

Since then, the strategy of cell fate conversion through transcription factor overexpression has been widely used in the generation of different types of stem cells and terminally differentiated cells from somatic cells. There are two approaches to achieving cell fate conversion, lineage-specific transcription factors and Yamanaka factors (Sox2, Klf4, Oct4, and c-Myc) [102–104]. Cardiomyocytes could be generated through overexpressing cardiac-specific transcription factor (Gata4, Mef2c, and Tbx5 or cardiac microRNAs) [102, 105–125] or Yamanaka factors in fibroblast cells [111, 123] (Supplementary Table 2).

Although the mechanism of cardiac cell fate conversion still remains unclear, genome-wide gene expression profile analysis showed that the cell fate conversion process induced cardiac gene expression and silenced the fibroblast-related genes. Direct cardiomyocyte generation from fibroblasts through cardiac-specific factors, such as GTM (Gata4, Tbx5, and Mef2c), did not pass a CPC stage [105, 109, 120, 125]. However, the combination of Sox2, Klf4, and Oct4 reprogrammed fibroblasts into cardiomyocytes through a CPC stage with Mesp1 and Isl1 expression [111, 123], indicating the possibility of generating induced CPCs from somatic cells *via* somatic programming.

## GENERATION OF INDUCED CARDIAC PROGENITOR CELLS VIA SOMATIC REPROGRAMMING

The reprogrammed cardiomyocytes have very low conversion efficiency and no proliferation capacity. Therefore, expansion *in vitro* for transplantation is not feasible. The therapeutic application of this strategy only relies on reprogramming *in vivo* [106, 108, 110, 114]. In addition to the low conversion efficiency *in vivo*, disturbing the function of cardiac fibroblasts with virus and gene overexpression might also affect the regenerative effects of cardiac fibroblasts that play a central role in heart remodelling and regeneration [126–128]. Therefore, it is necessary to generate CPC-like stem cells with the ability to regenerate the heart which might provide an unlimited source of stem cells with cardiac differentiation potentials [129–131] (Table 4).

We developed a novel approach to generate induced cardiospheres (iCS) from adult skin fibroblasts *via* somatic reprogramming [130]. After infection with Sox2, Klf4 and Oct4, iCS were generated from mouse adult skin fibroblasts treated with Gsk3β inhibitor-(2′Z, 3′E) - 6-Bromoindirubin-3′-oxime (BIO) and Oncostatin M. They resembled endogenous cardiospheres (eCS) with whole genome gene expression analysis, but contained a higher percentage of cells expressing Mesp1, Isl1 and Nkx2.5. They were differentiated into functional cardiomyocytes *in vitro* with similar electrophysiological properties, calcium transient and contractile function to eCS and mouse embryonic stem cell-derived cardiomyocytes. Transplantation of iCS into mouse myocardium following MI had similar effects to transplantation of eCS but significantly better than saline or fibroblast in improving left ventricular ejection fraction, increasing anterior/septal ventricular wall thickness and capillary density in the infarcted region 4 weeks after transplantation [130].

Zhang et al. also found that the induced CPC (Flk1^+^PdgfR⍺^+^ population) could be generated from mouse fibroblasts with overexpressing Sox2, Klf4, Oct4 and c-Myc plus BMP4, Activin A, CHIR99021 (GSK3beta inhibitor) and SU5402 (FGF, VEGF and PDGF inhibitor) stimulation [129]. The Flk1^+^PdgfR⍺^+^ induced CPC (iCPC) could be expandable and differentiated into cardiomyocytes, endothelial cells and smooth muscle cells. The differentiated cardiomyocytes had functional action potential and calcium transient activities. They could be stimulated by caffeine and isoproterenol. Whole genome expression analysis showed that they are similar with ESC derived CPCs. Transplanting Flk1^+^PdgfR⍺^+^ iCPC into MI mice model improved heart function [129].

On the other hand, the iCPC could be generated through lineage specific transcription factors Mesp1, Tbx5, Gata4, Nkx2.5 and Baf60c (MTGNB) plus LIF (leukemia inhibitory factor) and BIO stimulation [131]. Using a mouse model containing Nkx2.5-EYFP (enhanced yellow fluorescent protein) reporter system, the Nkx2.5-EYFP positive iCPC could be purified and expanded *in vitro* without expressing pluripotent markers. Whole genome expression analysis showed that they are similar with ESC derived CPCs. They could be differentiated into cardiomyocytes, endothelial cells and smooth muscle cells *in vitro* and cardiomyocytes *in vivo*. Transplanting these iCPC into MI mice model improved heart function without tumor formation [131].

## FUTURE PERSPECTIVES

The iCPC could be generated from somatic cells *via* somatic reprogramming strategy with lineage specific transcription factors or Yamanaka factors overexpression. The Nkx2.5^+^ and Flk1^+^PdgfR⍺^+^ iCPC are similar to mouse ESC derived CPCs, but whether they are also similar to endogenous CPCs or not remains unclear. The induced cardiosphere is similar to the endogenous cardiosphere but they both contain mixed cell populations, including CPCs and other supporting cells [132]. Therefore, more specific CPC surface markers and CPC purification methods should be developed. Furthermore, the iCPC are generated through a virus based method and the genome integration impairs their potential therapeutic applications. Thus integration-free methods should be applied in iCPC generation in the future.

## SUPPLEMENTARY MATERIALS TABLES


