# Supplementary material for: Generation of induced cardiac progenitor cells via somatic reprogramming
Source: Oncotarget. 2017 Feb 10;8(17):29442–57. doi: 10.18632/oncotarget.15272 (PMC5438743; doi:10.18632/oncotarget.15272)
Supplement: Supplementary file 1 [file oncotarget-08-29442-s001.docx]

| **Supplementary Table 1. Ongoing Clinical Trials (Further information could be found at https://clinicaltrials.gov)** | | | | | | | | | | | | | |
| --- | --- | --- | --- | --- | --- | --- | --- | --- | --- | --- | --- | --- | --- |
| **Trial Name/NCT Number** | **Trial Design** | | | | **Trial Phase** | **Cell Type** | | **No. of Patients Enrolled** | **Cell Delivery Method** | | **Trial Start Date** | | **Trial Status** |
| PERSEUS/ NCT01829750 | Randomized, Efficacy Study, Crossover Assignment | | | | Phase 2 | Cardiac Progenitor Cell | | 34 | Intracoronary | | April 2013 | | Active, not recruiting |
| CONCERT-HF/ NCT02501811 | Randomized, Safety/Efficacy Study, Parallel Assignment, Double Blind | | | | Phase 2 | c-kit+ Cardiac Progenitor Cell | | 144 | Transendocardial | | October 2015 | | Recruiting |
| NCT01758406 | Randomized, Safety/Efficacy Study, Parallel Assignment, Double Blind | | | | Phase 2 | Autologous Cardiac Progenitor Cell | | 50 | Intracoronary | | December 2013 | | Recruiting |
| CAREMI /NCT02439398 | Randomized, Safety/Efficacy Study, Parallel Assignment, Double Blind | | | | Phase 1 / Phase 2 | Autologous Cardiac Progenitor Cell | | 55 | Intracoronary | | June 2014 | | Active, not recruiting |
| TAC-HFT II/ NCT02503280 | Randomized, Safety/Efficacy Study, Parallel Assignment, Double Blind | | | | Phase 1 / Phase 2 | Autologous c-Kit Cardiac Progenitor Cell | | 55 | Intracoronary | | March 2020 | | Not yet recruiting |
| DYNAMIC/ NCT02293603 | Randomized, Safety Study, Single Group Assignment, Double Blind | | | | Phase 1 | Autologous Cardiosphere | | 42 | Intracoronary | | November 2014 | | Active, not recruiting |
| ACCRUE/ NCT01098591 | Case-Only, Retrospective | | | | N.A. | Cardiac Progenitor Cell | | 1000 | Intracoronary or Intramyocardial | | November 2007 | | Recruiting |
| ALLSTAR/ NCT01458405 | Randomized, Safety/Efficacy Study, Parallel Assignment, Double Blind | | | | Phase 1 / Phase 2 | Autologous Cardiosphere | | 134 | Intracoronary | | October 2012 | | Recruiting |
| **Supplementary Table 2. Cell Fate Conversion via Somatic Reprogramming in Cardiac Cells** | | | | | | | | | | | | | |
| **Factor Combination** | | **Species** | **Fibroblast Type** | **Characterization** | | | **Efficiency** | | | **Mechanism** | | **References** | |
| Gata4, Mef2c, Tbx5 | | Mouse | Cardiac; Dermal | Beating, calcium transient, action potential *in vitro*, CMC differentiation *in vivo* | | | 0.5% | | | Drive global gene expression to cardiomyocyte status | | 104 | |
| Adult ventricular CMC transcriptome | | Mouse | Embryonic | CMC marker expression, calcium transient, action potential | | | Rare | | | Not addressed | | 111 | |
| Gata4, Mef2c, Tbx5, Thymosin b4 | | Mouse | Cardiac | CMC generation *in vivo*, coupled action potential in *vivo*, cardiac function improved, | | | Rare | | | Not addressed | | 106 | |
| Gata4, Mef2c, Tbx5, Hand2 | | Mouse | Tail-tip; Cardiac | Cardiac function improved, calcium transient, contraction, action potential *in vivo* | | | 0.2% | | | Not addressed | | 107 | |
| Gata4, Mef2c, Tbx5, VEGF | | Rat | Dermal; Cardiac | CMC marker expression *in vitro*, improved heart function *in vivo* | | | Rare | | | Not addressed | | 113 | |
| Gata4, Mef2c, Tbx5 | | Mouse | Cardiac | CMC marker expression *in vivo* | | | Rare | | | Not addressed | | 105 | |
| Myocd, Mef2c, Tbx5, | | Mouse | Cardiac | CMC marker expression, CMC channel function, Sarcomeric organization | | | Rare | | | Not addressed | | 114 | |
| miR-1, miR-133, miR-208, miR-499 | | Mouse | Cardiac | CMC marker expression, sarcomeric organization, calcium transient *in vitro*, CM generation *in vivo*, heart function improvement | | | 1% | | | Not addressed | | 109; 123 | |
| Gata4, Mef2c, Tbx5, Myocd, Srf, Smarcd3, Mesp1 | | Mouse | Embryonic | CMC marker expression, CMC channel function | | | Rare | | | Not addressed | | 116 | |
| Gata4, Hand2, Myocardin, Tbx5, miR-1, miR-133 | | Human | Dermal; Cardiac | CMC marker expression, calcium transient, beating | | | Rare | | | Activation of cardiac genes and suppression of nonmyocyte genes | | 119 | |
| Gata4, Mef2c, ESRRB, Mesp1, ZFPM2, Myocardin, Tbx5 | | Human | Dermal; Cardiac | CMC marker expression, calcium transient, action potential | | | Rare | | | Not addressed | | 117 | |
| Gata4, Mef2c-MyoD fusion, Tbx5, Hand2 | | Mouse | Embryonic | CMC marker expression | | | Rare | | | Not addressed | | 118 | |
| Gata4, Mef2c, Mesp1, Myocardin, Tbx5 | | Human | Cardiac | CMC marker expression, calcium transient, action potential | | | Rare | | | Activation of cardiac genes and suppression of nonmyocyte genes | | 108 | |
| Gata4, Mef2c, Tbx5, Hand2, Nkx2.5 | | Mouse | Embryonic; Cardiac | CMC marker expression, calcium transient, beating | | | Rare | | | Not addressed | | 115 | |
| Gata4, Mef2c, Tbx5, miR-133 | | Mouse; Human | Embryonic; Cardiac | CMC marker expression, calcium transient, beating | | | Rare | | | Suppresses fibroblast genes and activate cardiac genes via Snai1 | | 121 | |
| ETS2, MESP1, | | Human | Dermal | CMC marker expression, calcium transient | | | Rare | | | Not addressed | | 112 | |
|  | |  |  |  | | |  | | |  | |  | |
| Gata4, Mef2c, Tbx5, Hand2, Nkx2.5, TGFb inhibition | | Mouse | Embryonic; Cardiac | CMC marker expression, calcium transient | | | Rare | | | Expression of cardiac genes by inhibition of TGFbeta | | 120 | |
| Mef2c-P2A-Gata4-T2A-Tbx5 | | Mouse | Cardiac; Tail-tip | CMC marker expression, calcium transient, beating | | | Rare | | | Enhanced expression of cardiac genes | | 124 | |
| Oct4, Sox2, Klf4 and c-Myc | | Mouse | Embryonic | CMC marker expression, beating | | | 40% | | | Through CPC stage | | 110 | |
| Oct4 | | Mouse | Embryonic; Tail-tip | CMC marker expression, beating | | | 0.5-1% | | | Through CPC stage | | 122 | |

CMC: cardiomyocyte
